# The Use of Water at Meals

**Published:** 1890-12

**Authors:** 


### THE USE OF WATER AT MEALS.

The *British Medical Journal* has this to say upon the matter of drinking freely of water at meals :

Opinions differ as to the effect of the free ingestion of water at meal times, but the view most generally received is probably that it dilutes the gastric juice and so retards digestion. Apart from the fact that a moderate delay in the process is by no means a disadvantage, as Sir William Roberts has shown in his explanation of the popularity of tea and coffee, it is more than doubtful whether any such effect is in reality produced. When ingested during meals, water may do good by washing out the digested food and by exposing the undigested part more thoroughly to the action of the digestive ferments. Pepsin is a catalytic body, and a given quantity will work almost indefinitely, provided the peptones are removed as they are formed.

The good effect of water drunk freely before meals, has, however, another beneficial result—it washes away the mucus which is secreted by the mucous membrane during the intervals of repose, and favors peristalsis of the whole alimentary tract. The membrane thus cleansed is in a much better condition to receive food and convert it into soluble compounds. The accumulation of mucus is especially well marked in the morning, when the gastric walls are covered with a thick, tenacious layer. Food entering the stomach at this time will become covered with this tenacious coating, which for a time protects it from the action of the gastric ferments, and so retards digestion. The tubular contracted stomach, with its puckered mucus lining and viscid contents, a normal condition in the morning before breakfast, is not suitable to receive food. Exercise before partaking of a meal stimulates the circulation of the blood, washes out the mucus, partially distends the stomach, wakes up peristalsis, and prepares the alimentary canal for the morning meal. Observation has shown that non-irritating liquids pass through the "tubular" stomach, and even if food be present, they only mix with it to a slight extent. According to Dr. Leuf, who has made this subject a special study, cold water should be given to persons who have sufficient vitality to react, and hot water to others. In chronic gastric catarrh it is extremely beneficial to drink warm or hot water before meals, and salt is said in most cases to add to the good effect produced.

